# Tamarind Seed (*Tamarindus indica*) Extract Ameliorates Adjuvant-Induced Arthritis *via* Regulating the Mediators of Cartilage/Bone Degeneration, Inflammation and Oxidative Stress

**DOI:** 10.1038/srep11117

**Published:** 2015-06-10

**Authors:** Mahalingam S. Sundaram, Mahadevappa Hemshekhar, Martin S. Santhosh, Manoj Paul, Kabburahalli Sunitha, Ram M. Thushara, Somanathapura K. NaveenKumar, Shivanna Naveen, Sannaningaiah Devaraja, Kanchugarakoppal S. Rangappa, Kempaiah Kemparaju, Kesturu S. Girish

**Affiliations:** 1 DOS in Biochemistry, University of Mysore, Manasagangothri, Mysore-570 006, India; 2 Department of Internal Medicine, Manitoba Centre for Proteomics and Systems Biology, University of Manitoba, Winnipeg- R3E3P4, Canada; 3 Department of Medical Biochemistry and Biophysics, Karolinska Institute, SE-171 77, Stockholm; 4 Applied Nutrition Discipline, Defence Food Research Laboratory, Mysore-570 011, India; 5 Department of Studies and Research in Biochemistry, Tumkur University, Tumkur-572 103, India; 6 DOS in Chemistry, University of Mysore, Manasagangothri, Mysore-570 006; India

## Abstract

Medicinal plants are employed in the treatment of human ailments from time immemorial. Several studies have validated the use of medicinal plant products in arthritis treatment. Arthritis is a joint disorder affecting subchondral bone and cartilage. Degradation of cartilage is principally mediated by enzymes like matrix metalloproteinases (MMPs), hyaluronidases (HAase), aggrecanases and exoglycosidases. These enzymes act upon collagen, hyaluronan and aggrecan of cartilage respectively, which would in turn activate bone deteriorating enzymes like cathepsins and tartrate resistant acid phosphatases (TRAP). Besides, the incessant action of reactive oxygen species and the inflammatory mediators is reported to cause further damage by immunological activation. The present study demonstrated the anti-arthritic efficacy of tamarind seed extract (TSE). TSE exhibited cartilage and bone protecting nature by inhibiting the elevated activities of MMPs, HAase, exoglycosidases, cathepsins and TRAP. It also mitigated the augmented levels of inflammatory mediators like interleukin (IL)-1β, tumor necrosis factor-α, IL-6, IL-23 and cyclooxygenase-2. Further, TSE administration alleviated increased levels of ROS and hydroperoxides and sustained the endogenous antioxidant homeostasis by balancing altered levels of endogenous antioxidant markers. Overall, TSE was observed as a potent agent abrogating arthritis-mediated cartilage/bone degradation, inflammation and associated stress *in vivo* demanding further attention.

Employing medicinal plants in the treatment of various human diseases is an ancient idea. In the due course, the recent and rigorous advancement in arthritis research has endorsed the use of medicinal plants in the disease treatment^1^. As known, arthritis is a degenerative joint disease affecting socio-economic life of middle-aged population. Recent survey by the World Health Organization reports that 10-15% of world and 15% of Indian population is arthritic and expected to double by 2030^2^. Arthritis is characterized by articular cartilage and subchondral bone degradation along with inflammation, immobility and pain. Articular cartilage, a specialized connective tissue containing chondrocytes surrounded by the components of extracellular matrix (ECM) such as collagen, hyaluronan (HA), aggrecan and other proteoglycans. The pathogenesis of arthritis involves enzymatic degradation of articular cartilage by matrix metalloproteinases (MMPs), hyaluronidases (HAase), aggrecanases and exoglycosidases. In addition, reactive oxygen species (ROS) and pro-inflammatory mediators like interleukin (IL)-1β, cyclooxygenase-2 (COX-2), IL-23 and tumor necrosis factor-α (TNF-α) and other cytokines contribute significantly by activating inflammatory signalling cascades^3^,^4^,^5^. Subsequently these events also promote osteoclasts to secrete bone resorbing enzymes like tartrate resistant acid phosphatase (TRAP), cathepsins and other phosphatases. The HA oligosaccharides and peptides, degradative end products of HA and collagen respectively, which are known to be pro-inflammatory in nature are also demonstrated to worsen the disease state. The oxidative stress during arthritis might have a gradual impact on blood components and vital organs, which is yet another reason to worry^6^. Thus, it is vital to inhibit cartilage/bone degeneration, inflammation and oxidative stress so as to regulate arthritis pathogenesis.

Nevertheless, the current preferred therapeutic drug treatments (NSAIDs and DMARDs) are reported with severe secondary ill effects in prolonged use. Thus, the use of medicinal plants is drawing special attention in the management of arthritis^7^. In view of this, the present study investigated anti-arthritic and anti-inflammatory abilities of tamarind seed extract (TSE) *in vivo*. Tamarind (*Tamarindus indica L.*) is a tree found in tropical and subtropical regions and has been considered as an important plant resource for food materials. It belongs to the family *Fabaceae* (Leguminosae) and the genus *Tamarindus*. It is cultivated mainly for the pulp in the fruit, which has been used in preparation of beverages, flavour confections, curries, and sauces and has also been preferred as an herbal medicine in various parts of the world. The salted and roasted seeds are also eaten in various parts of the world. Of late, tamarind seeds are receiving a lot of attention due to their therapeutic activities for plethora of human pathophysiological disorders including treatment of diabetes, snakebites, chronic diarrhoea, dysentery, jaundice, eye diseases, and ulcers^8^,^9^.

Previously we have reported the altered oxidative status of adjuvant induced arthritic rat liver specifically mitochondrial dysfunction and its amelioration by TSE^10^. Hence to further validate the anti-arthritic efficacy of TSE, the present study demonstrates the inhibition of cartilage and bone degrading factors (enzymatic and non-enzymatic) by TSE along with its composition. Furthermore, it also mitigates the augmented state of inflammation and oxidative stress by blocking over production of pro-inflammatory mediators and maintaining the homeostasis of endogenous antioxidant system.

## Results

### Identification of active components of TSE

**Figure 1:**
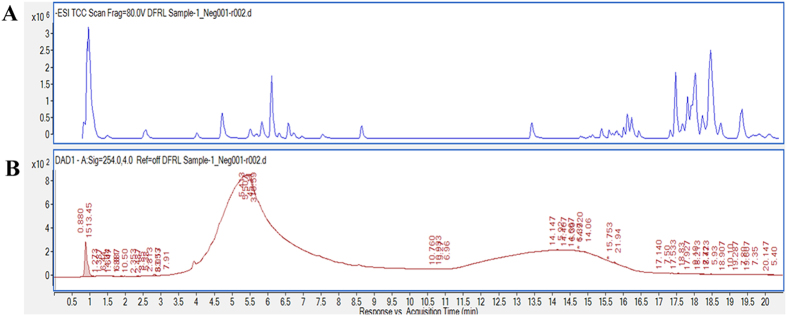
HPLC chromatogram of TSE. (**A**) Total ion current (TIC) chromatogram from HPLC separation of compounds present in the crude TSE extract and (**B**) HPLC-UV-Vis chromatogram of TSE at 280 nm.

The major compounds present in TSE were identified based on comparison of their retention times with those of reference compounds and their elution order on reverse phase C18 column. Peak assignment was confirmed by mass spectrometry. Further, molecular structure correlator (MSC) software was used to predict the possible structure for the obtained molecular formula and ESI-MS/MS fragmentation pattern (Supplementary Fig. S1-S6). Figure 1 represents the HPLC chromatogram of TSE showing total ion chromatogram and HPLC-UV-Vis chromatogram of the crude extract at 280 nm. Major compounds from the crude TSE were found to be Threo-Isocitric acid, Galactosyl glycerol, Procyanidin B2, Arecatannin B1, Catechin, Rutin and Embelin. (Supplementary Table S1).

### TSE reverts arthritis-induced physical changes

**Figure 2:**
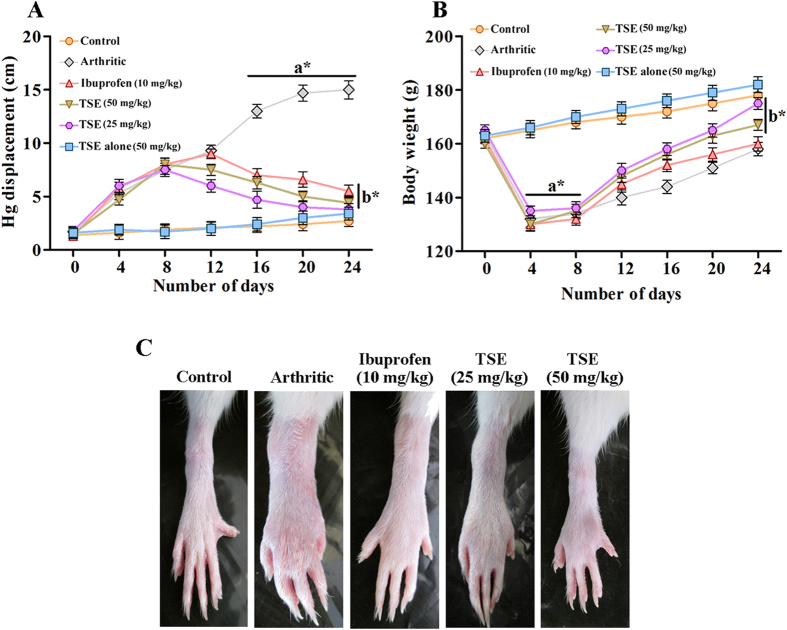
Influence of TSE on arthritis induced physical changes. (**A**) Measurement of paw edema by mercury displacement method. (**B**) Arthritis induced changes in body weight. (**C**) Macroscopic observation of severity of inflammation at paw joints. FCA injected day was considered as day zero, paw edema and body weight were measured until last day of treatment with an interval of every four days. From day eleven of FCA injection arthritic rats were treated with oral administration of TSE and Ibuprofen independently for fifteen days. Results are presented as mean ± SEM. a-compared with saline control and b-compared with arthritic group, * *p* < 0.05.

In the present study, we investigated the *in vivo* efficacy of TSE on Freund’s complete adjuvant (FCA) induced arthritis. In order to evaluate the anti-arthritic efficacy of TSE, paw swelling was evaluated by mercury displacement method. As paw swelling is one of the major factors in assessing the degree of inflammation and curative ability of anti-arthritic drugs. Oral administration of TSE (50 mg/kg) to arthritic rats showed a significant reduction in the paw volume than ibuprofen (10 mg/kg), an NSAID treatment control used in the study in comparison with arthritic group (Fig. 2A). A rapid reduction in the body weight was evident on the first week following the induction of arthritis to the animals and later a gradual increase in the body weight was observed on subsequent days. However, TSE treated groups showed a speedy recovery of body weight succeeding the treatment (Fig. 2B).

### TSE obstructs arthritis-induced hematological changes

Critical observation of hematocrit reveals the onset of arthritis induced severe hematological changes (Table 1). Arthritic group showed significant augmentation of WBC in comparison with saline control group. However, RBC and platelet count along with hemoglobin (Hb) levels were significantly reduced. Oral administration of TSE significantly restored the altered hematological changes induced due to arthritis progression.

### TSE improves arthritis-induced cartilage degradation

**Figure 3:**
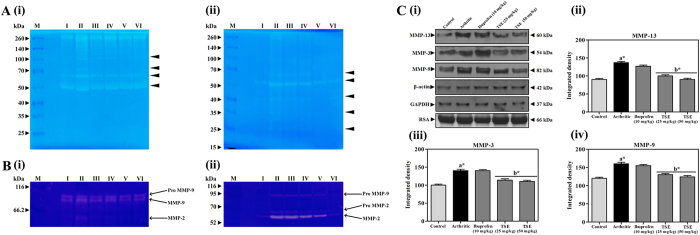
TSE alleviated cartilage degradation on arthritic rats. (**A**) Determination of hyaluronidase activity by zymography: (i) Serum hyaluronidase, (ii) Ankle bone joint hyaluronidase. Arrow head indicates the activity bands. M represents the molecular weight markers in kDa. (**B**) Determination of MMPs activity by zymography: (i) Serum MMPs, (ii) Ankle bone joint MMPs and the gels in this figure are cropped, full length gels are presented in supplementary figure S7. M indicates molecular weight markers in kDa. (**C**) Protein expression levels of serum MMPs measured by (i) Immunoblotting and their corresponding densitograms of (ii) MMP-13, (ii) MMP-3 and (iii) MMP-9. β-actin, GAPDH and rat serum albumin (RSA) were used as loading controls. Gel in this figure is cropped and the full length gel is presented in supplementary figure S7. Membrane was cut based on the molecular weight, probed with antibody of interest and band of interest were presented. Serum sample are as follows, Lane I – Saline treated, Lane II – Arthritic, Lane III – Arthritic rats treated with Ibuprofen (10 mg/kg), Lane IV – TSE treated arthritic rats (25 mg/kg), Lane V – TSE treated arthritic rats (50 mg/kg) and Lane VI – TSE alone (50 mg/kg). Results are presented as mean ± SEM. a-compared with saline control and b-compared with arthritic group, * *p* < 0.05.

**Figure 4:**
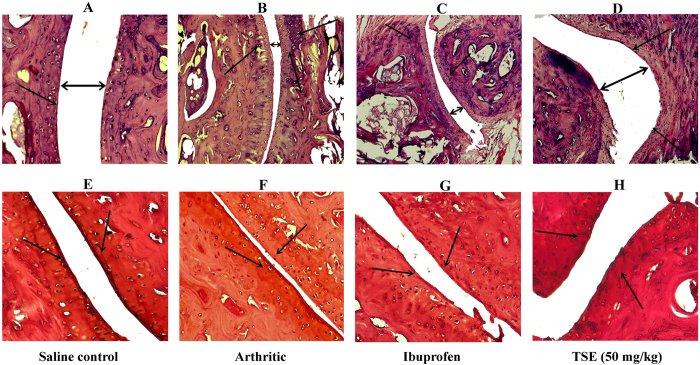
Histological analysis of Hematoxylin-eosin and Safranin-O stained right hind paw ankle joints from control and experimental groups of animals. Tissue sections from each group were stained with H & E (**A**–**D**) the arrows indicates, (**A**) Normal joint space with smooth and monolayer synovial cells lining of saline control joint. (**B**) Reduced joint space with inflammatory cells infiltration and moderate pannus formation of arthritic joint. (**C**) Moderately recovered joint space and pannus formation with inflammatory cells infiltration of ibuprofen treated joint. (**D**) Normal joint space with smooth synovial cell lining of TSE treated joint. Further, the glycosaminoglycan content in the knee joints of control and experimental groups were evaluated by Safranin-O staining (**E**–**H**), (**E**) Thick stained normal joints indicating high glycosaminoglycans content. (**F**), (**G**) Light stained arthritic and ibuprofen joints indicating deteriorated glycosaminoglycans and (**H**) Thick stained joints indicating the inhibition of glycosaminoglycans deterioration by TSE treatment. Original magnification 100x.

HAase and MMPs activities of serum and ankle joint bone homogenates in control and experimental groups were evaluated by zymography. The results revealed the augmented activity of HAase (Fig. 3A-i and -ii) and MMPs (Fig. 3B-i and -ii) in both serum and ankle joint homogenate of arthritic group with respect to saline control group. The augmented HAase and MMPs were indicated by clear translucent activity bands of arthritic group (lane-II) with respect to saline control group (lane-I). Further, immunoblots of MMP-13, -3 and -9 also revealed the elevated levels of MMPs in the arthritic group in comparison with saline control group (Fig. 3C i–iv). Oral administration of TSE effectively restored the augmented HAase and MMPs activities in arthritic rats, which clearly shows the ECM protective efficacy of TSE. In addition, histopathology of ankle joint of control and arthritic animals also confirmed the cartilage deterioration with reduced joint space and glycosaminoglycan content along with moderate pannus formation (Fig. 4). However, significant protection in the above parameters was apparent in the TSE treated group.

### TSE ameliorates bone degeneration

**Figure 5:**
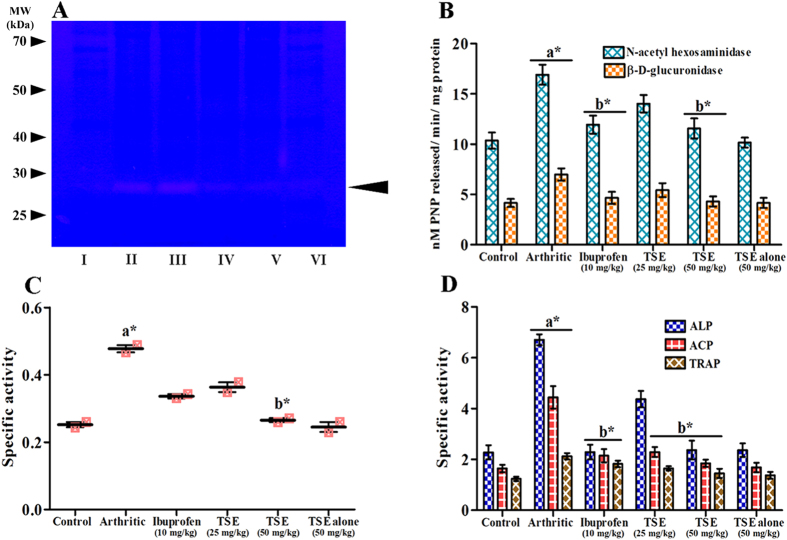
Effect of TSE on arthritis induced bone resorption. The right hind paw ankle bone joint homogenate of control and experimental animals were assessed for the bone resorption and degeneration. (**A**) Cathepsin K activity as determined by zymography. M indicates molecular weight markers in kDa. Arrow head indicates activity band (~37 kDa). The gel in this figure is cropped, full length gel is presented in supplementary figure S8. (**B**) Exoglycosidases activity. (**C**) Cathepsin D activity and (**D**) Alkaline phosphatase (ALP), acid phosphatase (ACP) and tartrate resistant acid phosphatase (TRAP) activities. Paw ankle joint sample are as follows, Lane I – Saline treated, Lane II – Arthritic, Lane III – Arthritic rats treated with Ibuprofen (10 mg/kg), Lane IV – TSE treated arthritic rats (25 mg/kg), Lane V – TSE treated arthritic rats (50 mg/kg) and Lane VI – TSE alone (50 mg/kg). Results are presented as mean ± SEM. a-compared with saline control and b-compared with arthritic group, * *p* < 0.05.

Protective efficacy of TSE on arthritis-induced bone degeneration was evaluated by assessing several bone degenerative enzymes on ankle joint bone homogenate. Cathepsin K, one of the potent mammalian collagenase has been implicated in various pathophysiological disorders of bone including arthritis. Cathepsin K (~37 kDa active form) appeared as zymographically active distinct band in arthritic group while, TSE treated groups abolished the activity and no such activity band was appeared in saline and TSE alone treated groups (Fig. 5A). Activities of *N*-acetyl hexosaminidase and β-D-glucuronidase in the arthritic group were found to be elevated by 64% and 68% respectively in comparison with saline control group. However, treatment with TSE (50 mg/kg) showed significant protection towards both the exoglycosidases by 88% and 96% and ibuprofen by 85% and 92% respectively in comparison with arthritic group (Fig. 5B). In addition, Cathepsin D (Fig. 5C), alkaline phosphatase (ALP), acid phosphatase (ACP) and TRAP (Fig. 5D) were also found to be significantly increased by 92%, 78%, 90% and 95% respectively compared to saline control group. TSE (50 mg/kg) treated group was shown to be significantly protective by diminishing the above enzyme activities by 89%, 89%, 93% and 90% respectively in comparison with arthritic group on the other hand ibuprofen offered protection up to an extent of 70%, 92%, 90% and 87% respectively.

### Ameliorative effect of TSE on inflammation and oxidative stress

#### **TSE mitigates arthritis-induced inflammatory mediators**

**Figure 6:**
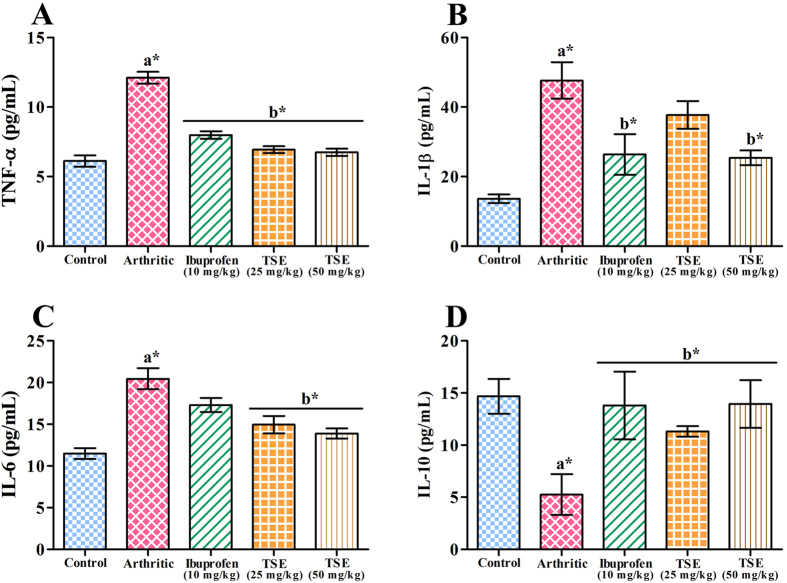
Protective role of TSE on inflammatory cytokines of control and experimental arthritic rats. Serum pro- and anti-inflammatory cytokines of experimental rats were quantified using ELISA kits as per manufacturer’s instructions. (**A**) TNF-α, (**B**) IL-1β, (**C**) IL-6 and (**D**) IL-10. Results are presented as mean ± SEM. a- compared with saline control and b-compared with arthritic group, * *p* < 0.05.

**Figure 7:**
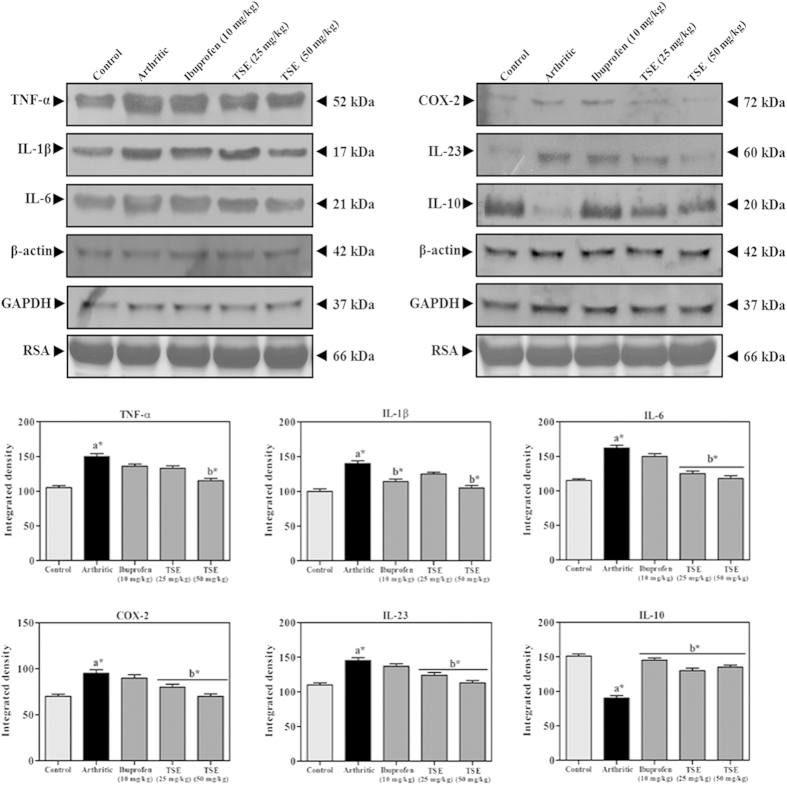
Influence of TSE on inflammatory mediators of control and experimental arthritic rats. Figure represents the protein expression levels of serum inflammatory mediators and corresponding densitograms of TNF-α, IL-1β, IL-6, COX-2, IL-23 and IL-10. Membrane was cut based on the molecular weight, probed with antibody of interest and band of interest were presented. β-actin, GAPDH and rat serum albumin (RSA) were used as loading controls. The gel in this figure is cropped, full length gel is presented in supplementary figure S9. Results are presented as mean ± SEM. a- compared with saline control and b-compared with arthritic group, * *p* < 0.05.

Inflammatory mediators are the key non-enzymatic factors involved in the cartilage degradation along with associated immunomodulation and oxidative stress. Ameliorative efficacy of TSE was evaluated on arthritis induced inflammation quantitatively by determining the serum levels of TNF-α, IL-1β, IL-6 and IL-10 using ELISA kits (Fig. 6). In addition the serum levels of TNF-α, IL-1β, IL-6, COX-2, IL-23 and IL-10 were also determined by immunobloting (Fig. 7). Both quantitative and immunoblot data revealed significant elevation in the levels of pro-inflammatory mediators such as COX-2, IL-1β, IL-6, and TNF-α and IL-23 in the arthritic group. In contrast, levels of anti-inflammatory cytokine IL-10 was significantly reduced in the arthritic group compared to saline control group. Oral administration of TSE (50 mg/kg) restored all the above inflammatory mediators to normal levels significantly in comparison with arthritic group. β-actin, GAPDH and rat serum albumin (RSA) were used as loading controls.

### TSE ameliorates arthritis-induced endogenous generation of ROS and hydroperoxides

**Figure 8:**
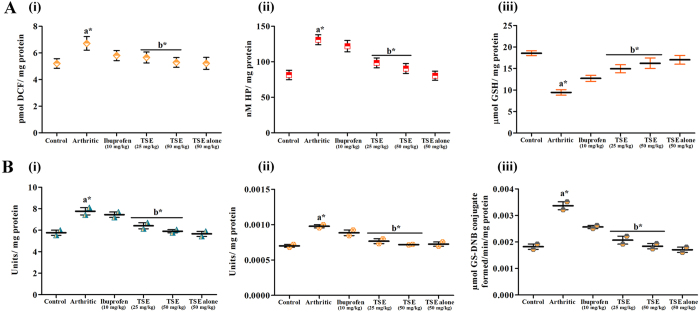
Effect of TSE on arthritis-triggered oxidative stress in control and experimental animals. (**A**) Influence of TSE on arthritis altered serum stress markers: (i) ROS, (ii) Hydroperoxides and (iii) GSH levels. (**B**) Influence of TSE on arthritis altered serum levels of endogenous antioxidant enzymes: (i) Superoxide dismutase, (ii) Catalase and (iii) Glutathione-*S*-transferase. Results are presented as mean ± SEM. a- compared with saline control and b-compared with arthritic group, * *p* < 0.05.

Onset of arthritis also induces stress in the affected individuals; the present study demonstrated the augmented levels of serum ROS and hydroperoxides of arthritic group significantly by 60% and 68% respectively in comparison with saline control group. Oral administration of TSE ameliorated the endogenously generated ROS and hydroperoxides by 98% and 89% and ibuprofen offered protection by 89% and 51% respectively in comparison with arthritic group (Fig. 8A i and ii).

### TSE administration sustains intracellular levels of GSH

Endogenous GSH is known to be the primary antioxidant molecule involved in the oxidation-reduction process and any alteration in the levels of GSH depicts the severity of oxidative stress on the system. The elevated levels of ROS directly affect the intracellular levels of GSH, hence there was a severe decline in the serum GSH pool of arthritic group by 47% with respect to saline control group. Oral administration of TSE rejuvenated the declined GSH by 91% and ibuprofen by 68% with respect to arthritic group (Fig. 8A iii).

### TSE abrogates augmented levels of endogenous antioxidant enzymes

The endogenous antioxidant enzymes remunerate the oxidative damage occurred during arthritic events. There was profound alteration in the levels of superoxide dismutase (SOD), catalase (CAT) and glutathione-*S*-transferase (GST) were observed. The experimental results revealed significant augmentation of serum SOD, CAT and GST of arthritic group by 74%, 82% and 70% respectively compared to saline control group. Oral administration of TSE revitalized the altered SOD, CAT and GST by 96%, 94% and 98% and ibuprofen fed group exhibited protection only by 40%, 56% and 65% respectively compared to arthritic group (Fig. 8B i-iii).

## Discussion

Treating arthritis has been an incessant challenge due to the severe side effects shown by NSAIDs and DMARDs. Thus, the use of medicinal plants has been regarded to be a better and efficient option, and also safe^7^. Use of different parts of tamarind tree for medicinal applications is well-documented in traditional medicine^8^. In view of this, the present study evaluated the anti-arthritic efficacy of TSE, considering cartilage and bone degeneration, inflammation and associated oxidative stress in FCA-induced arthritic rats. This is the first study to evaluate the anti-arthritic efficacy of TSE.

Upon adjuvant injection, the physical changes like decreased body weight, paw swelling, exhibiting tapering of bone joint were evident in arthritic rats confirming the disease perpetuation in the rats. The oral administration of TSE effectively reduced paw swelling and aided body weight regain. Further, TSE significantly protected cartilage and bone degeneration by alleviating augmented levels of cartilage degrading enzymes like HAase and MMPs, and bone resorbing enzymes such as, exoglycosidases, TRAP, cathepsins, ACP and ALP. The enzymes HAase and MMPs are primarily demonstrated to degrade HA and collagen respectively, the backbones of articular cartilage. Several studies have reported the increased activities of these enzymes, both in serum and synovial fluid of arthritic patients^11^,^12^. Similarly, increased activities of β-D-glucuronidase, *N*-acetyl-hexosaminidase, TRAP and cathepsins (K and D) were shown in the serum and synovial joint of arthritic individuals contributing to reduced bone formation and an increased bone resorption during arthritis. The increased activity of HAase was known to release pro-inflammatory HA fragments that in turn activate exoglycosidases, which contribute to cartilage and bone degeneration by clearing the HA fragments^13^. This can also end up in triggering TRAP and cathepsins the key regulators of bone resorption and deformity.

Besides, the ROS and the pro-inflammatory mediators are defined to be highly intimidating key non-enzymatic factors accountable for the progression of arthritis or even making it worse by eliciting immunological cell activation and signaling cascade^14^. Several studies have reported the increased levels of ROS and pro-inflammatory mediators during arthritis. Further, TNF-α and IL-1β are also known as potent stimulators of mesenchymal cells such as, synovial fibroblasts, osteoclasts, and chondrocytes that release tissue-destroying MMPs^15^,^16^. From the results, TSE impedes augmented generation of endogenous ROS and hydroperoxides efficiently in serum and it is noteworthy to mention it is far better than standard drug ibuprofen. Further, TSE significantly mitigates augmented serum levels of TNF-α, IL-1β, IL-6, COX-2 and IL-23, on the other hand, TSE was able to elevate the levels of IL-10, an anti-inflammatory mediator. These activities exhibited by TSE are majorly attributed to its antioxidant, anti-inflammatory and free radical scavenging properties.

Rigorous and continuous production of ROS and pro-inflammatory mediators were shown to further trigger oxidative stress in the arthritic individuals^17^. As a consequence, altered levels of GSH, the first line of defence in endogenous antioxidant system and other antioxidant enzymes have been sighted^18^. The current results also demonstrated the depletion of GSH levels in the course to withstand the oxidative damage. Furthermore, arthritic group was observed with augmented activities of endogenous GST, SOD and CAT antioxidant enzymes. Several studies have demonstrated elevated levels of endogenous antioxidant enzyme activities during ROS triggered oxidative stress^19^. TSE supplementation efficiently controlled the oxidative flux and aided the maintenance of the homeostasis of endogenous antioxidant system by balancing the levels of GSH, SOD, CAT and GST. Besides, the severe stress also resulted in altered number of blood components due to the continuous secretion of ROS and oxidation of circulatory proteins. The increased WBC count and decreased RBC and platelet count along with reduced levels of Hb in arthritic rats depicts the circulatory blood stress^20^. TSE treatment successfully balanced the Hb levels and WBC, RBC and platelet count.

The qualitative LC-MS analysis of TSE revealed the presence of procyanidin B2, catechin, rutin, embelin, arecatannin B1, D-threo-isocitric acid and galactosyl glycerol. Procyanidin B2, catechin, rutin and embelin have been reported to be potent antioxidant and anti-inflammatory molecules, which can suppress oxidative stress^21^,^22^,^23^. Further, catechin and rutin are known to inhibit MMPs and HAase responsible for cancer metastasis and matrix degradation^13^,^24^. All these molecules are shown to modulate MAP kinase-mediated pro-inflammatory and NF-κB pathways effectively. Hence, TSE is a cocktail of all good therapeutics, which can bring an efficient combinatorial effect on an ailing system.

## Conclusion

**Figure 9:**
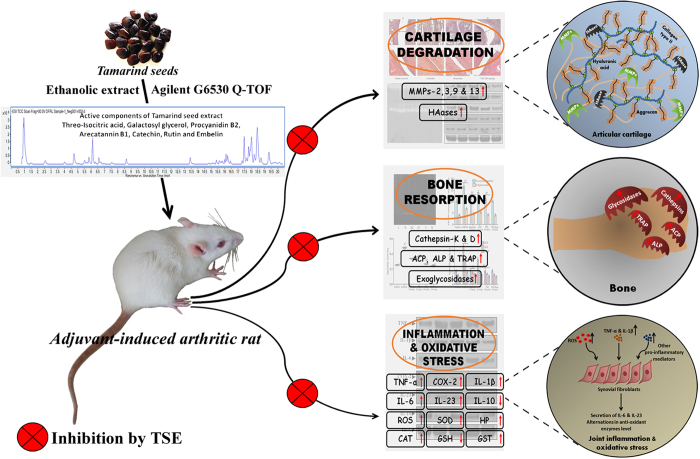
Proposed mechanism of action of anti-arthritic efficacy of Tamarind seed extract (TSE). TSE is demonstrated to block arthritis-mediated cartilage degeneration, bone resorption, inflammation and oxidative stress by modulating the respective enzymatic and non-enzymatic parameters. The rat picture used in the figure is captured by Mr. MS Sundaram (first author) while performing animal experiment for the present study.

Tamarind is a tropical tree, which has been considered as an important food plant. However, it has also been preferred as an herbal medicine in various parts of the world. Of late, tamarind seeds have received much attention due to their therapeutic activities for plethora of human pathophysiological disorders including treatment of diabetes, snakebites, chronic diarrhoea, dysentery, jaundice, eye diseases, and ulcers. The present study, demonstrated the abrogation of cartilage and bone degrading factors (enzymatic and non-enzymatic) by TSE. It also inhibited the augmented status of inflammation and oxidative stress by modulating elevated levels of pro-inflammatory mediators and by balancing the homeostasis of endogenous antioxidant system. In addition, TSE successfully regulated the Hb levels and circulatory WBC, RBC and platelet count. Altogether, TSE was shown to be a potent anti-arthritic, anti-inflammatory and anti-stress agent (Fig. 9).

## Materials and methods

### Chemicals

Freund’s complete adjuvant (FCA) containing 10 mg/mL heat-killed *Mycobacterium tuberculosis*, dihydrodichlorofluorescein diacetate (DCFDA), *O*-phthalaldehyde (OPT), HEPES and 1,1′,3,3′-tetramethoxypropane (TMP), dithiothreitol (DTT), dichlorofluorescein (DCF), hyaluronic acid sodium salt, gelatin (Type A from porcine skin), *p*-nitrophenyl β-*N*-acetyl glucosaminide, *p*-nitrophenyl β-glucuronide, reduced glutathione (GSH) and hemoglobin were procured from Sigma Chemicals, St. Louis, USA. TNF-α, IL-6 and IL-1β antibodies were from Cayman Chemicals, Michigan, USA, MMP-13, -3, -9, COX-2 and β-actin antibodies were from Epitomics, Inc. Burlingame, USA, IL-10 and IL-23 antibodies were from Thermo Fisher Scientific, New Hampshire, USA. GAPDH antibody was from were from Cell Signalling and Technology, Beverly, USA. Mini ELISA development kits for IL-6, IL-1β, TNF-α, and IL-10 were obtained from PeproTech, KHC Healthcare, India. Tamarind seeds were from local market, Mysore. All other chemicals used in this study were of analytical grade, purchased from Sisco Research Laboratories, Mumbai, India.

### Experimental animals and study design

Adult Wistar rats (10 weeks old) obtained from Central Animal Facility (University of Mysore, Mysore) were used in the present study. The animals were divided into 6 groups consisting of 6 animals per group (n = 6). Group I- Saline control rats; Group II- Arthritic rats; Group III- Arthritic rats orally administered with Ibuprofen drug (10 mg/kg/day); Group IV- Arthritic rats orally administered with TSE (25 mg/kg/day); Group V- Arthritic rats orally administered with TSE (50 mg/kg/day); Group VI- Rats orally administered with TSE alone (50 mg/kg/day).

### Animal Ethical Statement

The animal experiment was reviewed and approved by the Institutional Animal Ethical Committee (Letter No. UOM/IAEC/08/2013; Dated 28-09-2013) University of Mysore, Mysore. Animal handling and experiments were carried out in accordance with the guidelines of the Committee for the Purpose of Control and Supervision of Experiments on Animals (CPCSEA).

### Inducing arthritis in Wistar rats

Arthritis was induced by subcutaneously injecting 100 μL FCA at the back surface of right hind paw. The symptoms of arthritis were observed within 10 days of FCA injection in all animals except group I and VI. The respective treatments were begun from day 11 and treated every day for next 15 days. The treatment dosage of TSE was fixed based on our preliminary results. The animals were euthanized on day 26 and blood was obtained *via* cardiac puncture. After allowing the blood to clot, serum was separated by centrifuging it at 2000 rpm for 15 min and the collected serum was stored at −80 °C until further use. The right hind paw ankle joints were collected from all animals and stored at −80 °C for the assessment of cartilage degradation and bone resorptive enzymes.

### Tamarind seed extract (TSE) preparation

TSE was prepared as described previously^25^. The saline dissolved TSE was used in further experiments.

### Qualitative LC-MS analysis of TSE

TSE was analysed on the LCMS-IT-TOF using the conditions found in supplementary Table 2. Agilent G6530 Q-TOF equipped with Agilent Jet Stream source used for the identification of active components in the extract. The obtained Q-TOF data was processed using molecular feature extraction (MFE) algorithm and Metlin data base.

### Physical assessment

Arthritis severity was assessed by the degree of edema induced at the paw after FCA injection. On every four alternate days paw volumes of all the rats were measured by mercury displacement method. The difference in paw weight was statistically expressed as centimetres of mercury displaced^26^. The body weight of all the rats were measured on every four alternate days. The difference in body weight was statistically expressed in grams and the graph was plotted.

### Hematological studies

A portion of blood was mixed with anticoagulant (2.5% tri sodium citrate, 1.37% citric acid and 2% glucose in the ratio 1:5; anticoagulant : blood, v/v) and was used to assess various hematological parameters *viz*., total leukocyte count (WBC), total erythrocyte count (RBC), hemoglobin content (Hb) and platelet count were determined using an automated haematology analyser (Sysmex KX-21, Japan)

### Assessment of cartilage degradation

#### Determination of HAase activity

HAase activity was evaluated according to the method of Girish *et al.*^27^ in serum samples and right hind paw ankle joint homogenate of control and experimental animals. HAase activity was detected as unstained translucent bands against blue back ground.

#### Determination of MMPs activity

MMPs activity was evaluated by zymography as described by La Rocca *et al.*^28^ in serum samples and right hind paw ankle joint homogenate of control and experimental animals. MMPs activity was detected as unstained translucent bands against dark blue back ground.

#### Estimation of MMPs

The serum levels of MMP-13, MMP-3 and MMP-9 were estimated by immunobloting. Serum samples (50 μg protein) were electrophoresed on SDS-PAGE (10%), and the separated bands were transferred onto PVDF membranes using wet-blot unit (50 V for 50 min). After transfer, membrane was cut based on the molecular weight and blocked with 5% non-fat milk powder dissolved in Tris-buffered saline containing Tween-20 (TBST, 10 mM Tris-HCl pH 8.0, 150 mM NaCl, 0.05% Tween-20). The blots were then probed with respective antibodies. Further, the blots were developed using enhanced chemiluminescence (ECL) western blot detecting reagent. Blots were incubated with the detection reagent for 1 min and then exposed to blue sensitive X-ray film for 1, 2 and 5 min. The films were developed using radiograph developer. β-actin, GAPDH and rat serum albumin (RSA) were used as loading controls. For re-probing, blots were incubated in stripping buffer (200 mM glycine, pH-2.2, 1% Tween-20 and 0.1% SDS) for 2 min and washed with TBST. Blots were again treated with stripping buffer for 5 min and washed thrice with TBST, 2 min each wash. The stripped membrane was blocked in TBST containing 5% non-fat milk powder over night at 4 °C and probed with desired antibodies. Each membrane was stripped three times maximum.

### Assessment of bone degeneration

#### Determination of bone metabolic enzyme activities

The degree of bone degeneration and resorption was assessed by measuring bone metabolic enzymes and Cathepsins activity in ankle paw joint homogenate. The ankle joints (200 mg) from right hind paw of experimental and control animals were obtained by removing skin and muscle, crushed with a mortar and pestle and homogenized at 4 °C in 100 mM potassium phosphate buffer (pH 7.4). Further the paw homogenates were spun at 8000 rpm for 15 min at 4 °C. In the supernatant, Cathepsin K activity of control and experimental animals was evaluated according to the method of Li *et al.*^29^. Cathepsin K activity was detected as unstained translucent bands against blue back ground. β-*N*-acetylhexosaminidase and β-D-glucuronidase activities were measured by determining the *p*-nitrophenol released from *p*-nitrophenyl β-*N*-acetyl glucosaminide and *p*-nitrophenyl β-glucuronide, respectively^30^. Further, ACP, TRAP^31^ and ALP^32^ activities were measured using *p*-nitrophenyl phosphate as the substrate and expressed as nM *p*-nitrophenol released/min/mg protein. Cathepsin D activity was measured according to the protocol of Folin and Ciocalteau^33^ using hemoglobin as substrate. Briefly, the tissue homogenate (100 mg) was added to 100 mM sodium acetate buffer (pH 4.0) and incubated with hemoglobin (2%, w/v) for 2.5 h at 37 °C. The enzymatic reaction was stopped by adding 1 mL of 10% trichloroacetic acid and centrifuged at 2500 rpm for 10 min. The released peptides in the supernatant were measured using Folin Ciocalteu’s reagent at 660 nm. The amount of enzyme required to increase an absorbance of 0.01 at 660 nm/min at 37 °C is considered as one unit of activity. Specific activity was expressed as units/min/mg protein.

### Assessment of Inflammation and Oxidative stress

#### Estimation of inflammatory mediators

The serum levels of inflammatory mediators like TNF-α, IL-1β, IL-6 and IL-10 were quantitatively estimated by using ELISA kits as per the manufacturer’s protocol. In addition, immunobloting was carried out to estimate the serum TNF-α, IL-1β, IL-6, COX-2, IL-23 and IL-10. β-actin, GAPDH and rat serum albumin (RSA) were used as loading controls.

#### Estimation of reactive oxygen species

The endogenous generation of ROS in the serum of all groups was quantified by the method of Driver *et al.*^34^ with slight modification using DCFDA, a non-polar compound that, after conversion to its polar derivative by intracellular esterases, rapidly reacts with ROS to form a highly fluorescent compound DCF. Serum ROS levels were expressed as pmol DCF formed/mg protein.

#### Estimation of hydroperoxides

The hydroperoxides in the serum was quantified using homovanillic acid, a specific H_2_O_2_-sensitive fluorescent probe by following the method of Botsoglou *et al.*^35^ The levels of hydroperoxides were expressed as nmol hydroperoxide (HP)/mg protein.

#### Serum GSH Measurement

GSH content in serum was measured as described by Mokrasch and Teschke^36^ with slight modifications. The GSH content was represented as nmol GSH/mg protein.

#### Determination of stress marker enzymes

Superoxide dismutase activity in serum was measured by supervising the quercetin autoxidation inhibition^37^. Similarly, Catalase activity in serum was determined by monitoring the rate of H_2_O_2_ hydrolysis at 240 nm and expressed as mol H_2_O_2_ decomposed/min/mg protein^38^. In contrast, GST activity was measured by measuring the amount of enzyme catalysed conjugation of GSH with 1-chloro-2,4-dinitro benzene (CDNB) at 340 nm and expressed as μmol conjugate formed/min/mg protein^39^.

#### Histological assessment of inflammation

Dissected rat ankles were fixed in 10% buffered formalin followed by decalcification using 10% EDTA and processed for paraffin embedding. Tissues were sectioned (5 μm) using microtome and subsequently stained with hematoxylin-eosin (H and E) and Safranin-O staining and photographed using Axio imager.A2 microscope.

#### Determination of protein concentration

Protein concentration was estimated by following the protocol of Lowry *et al.*^40^

### Statistical analysis

Statistical analysis was performed using GraphPad Prism statistical software. All of the results were represented as mean ± SEM of three independent experiments. To assess the differences between the groups One-way ANOVA was carried out and followed by Tukey “honestly significantly different” (HSD) *post hoc* analysis. A *p* value of <0.05 was considered to be significant.

## Additional Information

**How to cite this article**: Sundaram, M. S. *et al.* Tamarind Seed (*Tamarindus indica*) Extract Ameliorates Adjuvant-Induced Arthritis *via* Regulating the Mediators of Cartilage/Bone Degeneration, Inflammation and Oxidative Stress. *Sci. Rep.* **5**, 11117; doi: 10.1038/srep11117 (2015).

## Supplementary Material

Supplementary Information

## Figures and Tables

**Table 1:** Altered hematological status of control and experimental arthritic rats.

	**Groups**			
**Parameters**	**Control**	**Arthritic**	**Ibuprofen administered (10** **mg/kg)**	**TSE administered (50** **mg/kg)**
**Total WBC count (x10**^**3**^**/μL)**	2.7 ± 0.8	7.5 ± 0.6*	5.5 ± 0.7	3.3 ± 0.8†
**RBC count (x10**^**6**^**/ μL)**	5.6 ± 0.3	3.0 ± 0.5*	4.8 ± 0.4†	4.9 ± 0.8^†^
**Hemoglobin (g/dL)**	8.2 ± 0.7	4.7 ± 0.4*	5.1 ± 0.5	6.4 ± 0.3†
**Platelet count (x10**^**3**^**/ μL)**	560 ± 4.3	317 ± 3.1*	344 ± 2.8	485 ± 4.2†

Results are presented as mean ± SEM.

^*^- compared with saline control and.

^†^- compared with arthritic group, *,† p < 0.05.
