# Supplementary material for: Tamarind Seed (Tamarindus indica) Extract Ameliorates Adjuvant-Induced Arthritis via Regulating the Mediators of Cartilage/Bone Degeneration, Inflammation and Oxidative Stress
Source: Sci Rep. 2015 Jun 10;5:11117. doi: 10.1038/srep11117 (PMC4461917; doi:10.1038/srep11117)
Supplement: Supplementary Information [file srep11117-s1.pdf]

Tamarind Seed (*Tamarindus indica*) Extract Ameliorates Adjuvant Induced Arthritis *via* Regulating the Mediators of Cartilage/Bone Degeneration, Inflammation and Oxidative Stress

Mahalingam S. Sundaram[1], Mahadevappa Hemshekhar[1,2], Martin S. Santhosh[1,3], Manoj Paul[1], Kabburahalli Sunitha[1], Ram M. Thushara [1], Somanathapura K. NaveenKumar[1], Shivanna Naveen[4], Sannaningaiah Devaraja[5], Kanchugarakoppal S. Rangappa[6]*, Kempaiah Kemparaju[1]*, Kesturu S. Girish[1,5]*

[1]DOS in Biochemistry, University of Mysore, Manasagangothri, Mysore-570 006, India; [2] Department of Internal Medicine, Manitoba Centre for Proteomics and Systems Biology, University of Manitoba, Winnipeg-R3E3P4, Canada; [3]Department of Medical Biochemistry and Biophysics, Karolinska Institutet, SE-171 77, Stockholm;[4]Applied Nutrition Discipline, Defence Food Research Laboratory, Mysore-570 011, India; [5]Department of Studies and Research in Biochemistry, Tumkur University, Tumkur-572 103, [6]DOS in Chemistry, University of Mysore, Manasagangothri, Mysore-570 006; India.

*** Corresponding authors**

Address of correspondence

1) **Dr. Kesturu S. Girish**
   Associate Professor
   Department of Studies and Research in Biochemistry
   Tumkur University, Tumkur-572 103, Karnataka INDIA
   University of Mysore, Mysore-570 006, Karnataka, INDIA
   E-mail: ksgbaboo@gmail.com Tel.: +91-9964080540
2) **Dr. Kempaiah Kemparaju**
   Professor
   DOS in Biochemistry, University of Mysore, Karnataka, INDIA
   E-mail: kemparajuom@gmail.com Tel.: +91-9945996543
3) **Dr. Kanchugarakoppal S. Rangappa**
   Professor
   DOS in Chemistry, University of Mysore, Karnataka, INDIA
   E-mail: rangappaks@gmail.com Tel.: +91-821-2419666

Supplementary Information

Supplementary Tables:

Table S1

Composition of major compounds present in crude TSE analysed using LC-MS-IT-TOF

Sl No.	Label	Name	Formula	Score	Mass	Avg Mass	Mass (DB)	m/z	RT
1	Cpd 1	Threo-Isocitric acid	$C_6H_8O_7$	99.66	192.026	192.118	192.027	191.019	0.847
2	Cpd 3		$C_{25}H_{44}O_{21}$	99.68	680.237	680.611		679.230	0.909
3	Cpd 4		$C_{19}H_{34}O_{16}$	99.57	518.184	518.446		517.170	0.913
4	Cpd 13	Galactosyl glycerol	$C_9H_{18}O_8$	99.53	254.100	254.232	254.100	253.092	0.954
5	Cpd 18	Maltose	$C_{12}H_{22}O_{11}$	99.74	342.116	342.308	342.116	341.109	0.979
6	Cpd 23		$C_{14}H_{28}O_{15}S$	99.49	468.114	468.436		467.107	1.081
7	Cpd 27		$C_{18}H_{27}NO_{14}$	99.56	481.142	481.398		480.135	2.564
8	Cpd 28		$C_{17}H_{16}N_5O_{16}$	98.80	546.059	546.308		545.051	2.565
9	Cpd 31	Procyanidin B2	$C_{30}H_{26}O_{12}$	99.44	578.142	578.509	578.520	577.135	4.726
10	Cpd 32	Arecatannin B1	$C_{45}H_{38}O_{18}$	99.54	866.205	866.760	866.772	865.198	5.514
11	Cpd 33		$C_{60}H_{50}O_{24}$	99.42	1154.268	1154.992		1153.261	5.696
12	Cpd 34	Catechin	$C_{15}H_{14}O_6$	99.71	290.079	290.271	290.079	289.071	5.845
13	Cpd 35		$C_{26}H_{40}O_{12}$	99.49	544.252	544.585		543.245	6.111
14	Cpd 36		$C_{26}H_{40}O_{12}$	99.05	544.252	544.576		543.244	6.326
15	Cpd 38		$C_{14}H_{18}O_9$	99.50	330.094	330.276		329.087	6.740
16	Cpd 40		$C_{30}H_{26}O_{12}$	99.25	578.142	578.510		577.135	7.550
17	Cpd 41		$C_{27}H_{39}NO_{14}$	99.64	601.237	601.607		600.607	8.640
18	Cpd 43	Rutin	$C_{27}H_{30}O_{16}$	98.98	610.507	610.507	610.513	609.146	8.651
19	Cpd 44	Embelin	$C_{17}H_{26}O_4$	99.63	294.182	294.383	294.183	293.175	13.432

The major compounds present in the crude TSE were analysed on LC-MS-IT-TOF by using Agilent G6530 Q-TOF equipped with Agilent jet stream source. Identification of active components in the extract was confirmed by their retention time with those of reference compounds and elution order on reverse phase C18 columns. DB-Data Base; m/z-mass/charge ratio; RT-Retention Time (min).

Table S2

Analytical Conditions for LCMS-IT-TOF

**LC parameters**	
Column	2.1 mm x 150 mm, Eclipse Plus C18 1.8 µm
Mobile phase A	H_2O 10 mM ammonium acetate
Mobile phase B	Methanol
LC Time program (linear gradient)	10% B (0-1 min); 10-30% B (1-5 min); 30-90% B (5-10 min); 90% B (10-18 min); 90-10% B (18-18.5 min)
Flow rate	0.3 ml/min
Injection volume	2.0 µL
**MS parameters**	
ESI Positive	2 GHz mode
Scan Range	100-1500 m/z
Scan Rate	2 Hz
Reference ions	112.9855 m/z, 1033.9881 m/z
**Source parameters**	
Drying Gas Temp	350 °C
Drying Gas Flow	7 l/min
Nebulizer	50 p.s.i.
Sheath Gas Temp	400 °C
Sheath Gas Flow	11 l/min
VC cap	4500 V
Fragmentor	80 V
Nozzle voltage	0 V

Qualitative LC-MS Analysis was performed on LC-MS-TOF using Agilent G6530 Q-TOF

Supplementary figures

Supplementary Figure S1

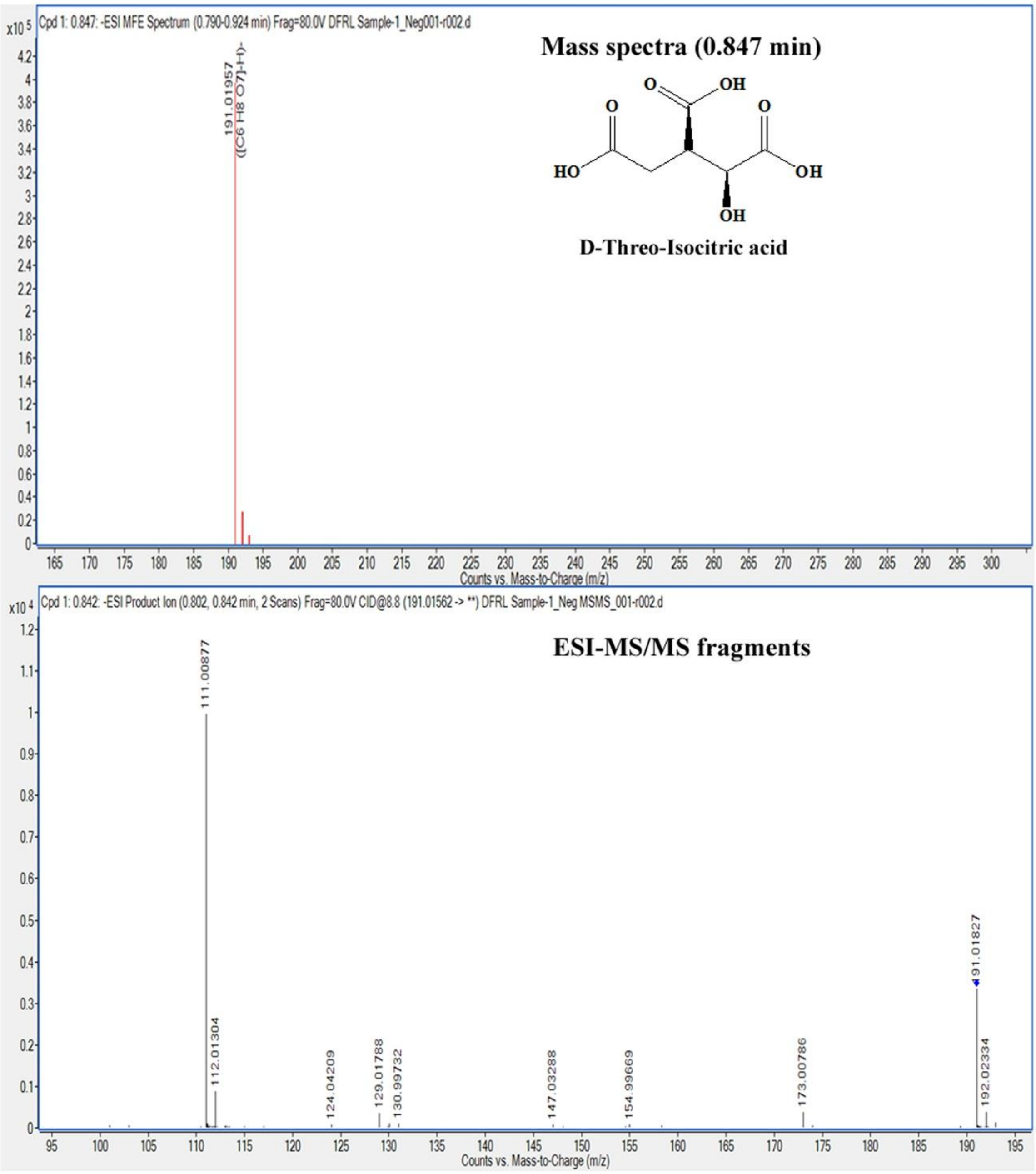

Supplementary Figure S1. LC-MS and ESI-MS/MS profile of TSE showing D-Threo-Isocitric acid. Peak was obtained at retention time 0.847 minutes. Signal obtained in ESI-MS/MS corresponds to its absolute mass m/z 192.027 daltons. Molecular structure correlator (MSC) software was used to predict the possible structure for the obtained molecular formula and fragmentation pattern. Chemical structure of D-Threo-Isocitric acid was drawn by using Chem Draw software.

Supplementary Figure S2

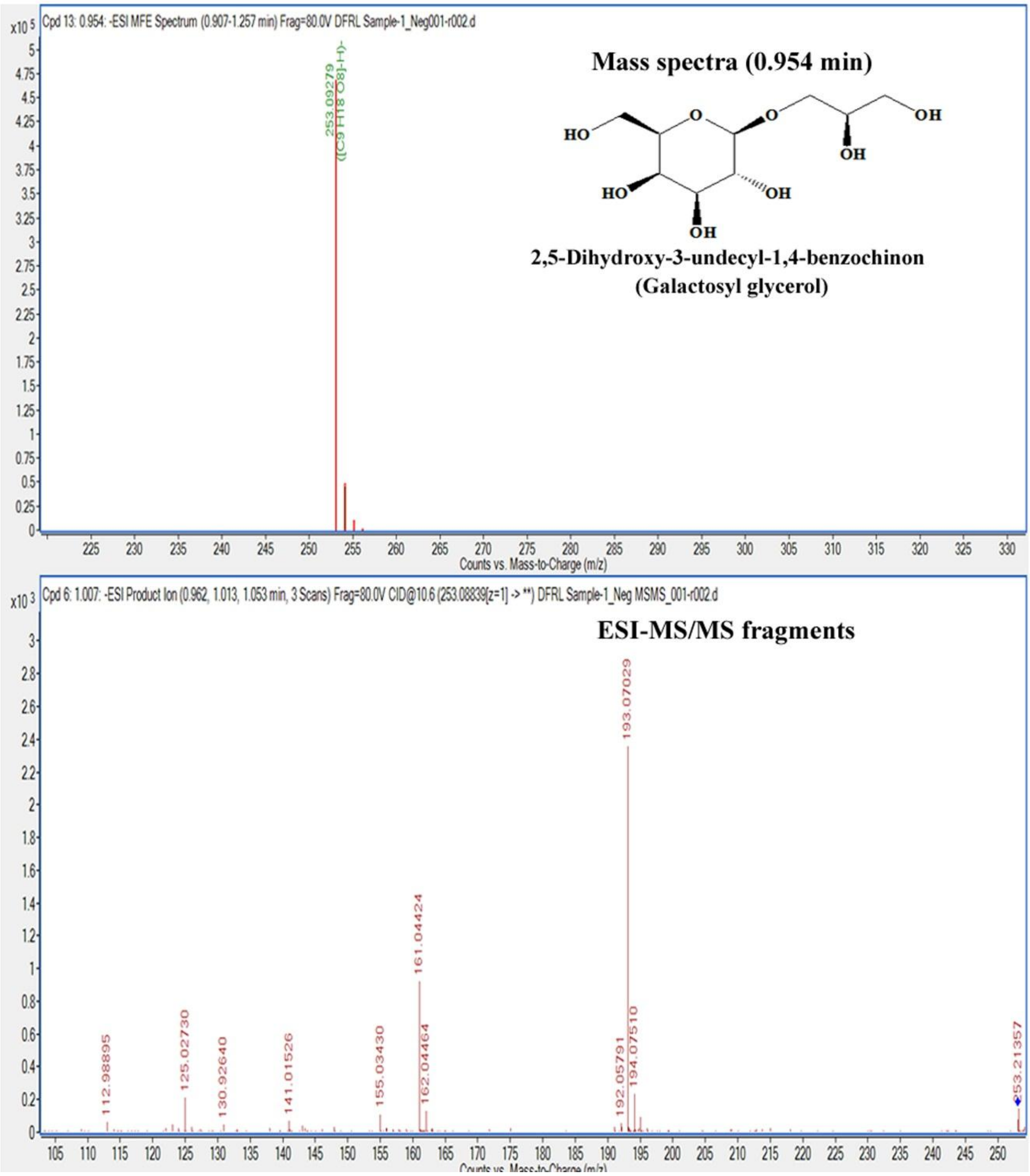

Supplementary Figure S2. LC-MS and ESI-MS/MS profile of TSE showing Galactosyl glycerol. Peak was obtained at retention time 0.954 minutes. Signal obtained in ESI-MS/MS corresponds to its absolute mass m/z 254.100 daltons. Molecular structure correlator (MSC) software was used to predict the possible structure for the obtained molecular formula and fragmentation pattern. Chemical structure of Galactosyl glycerol was drawn by using Chem Draw software.

Supplementary Figure S3

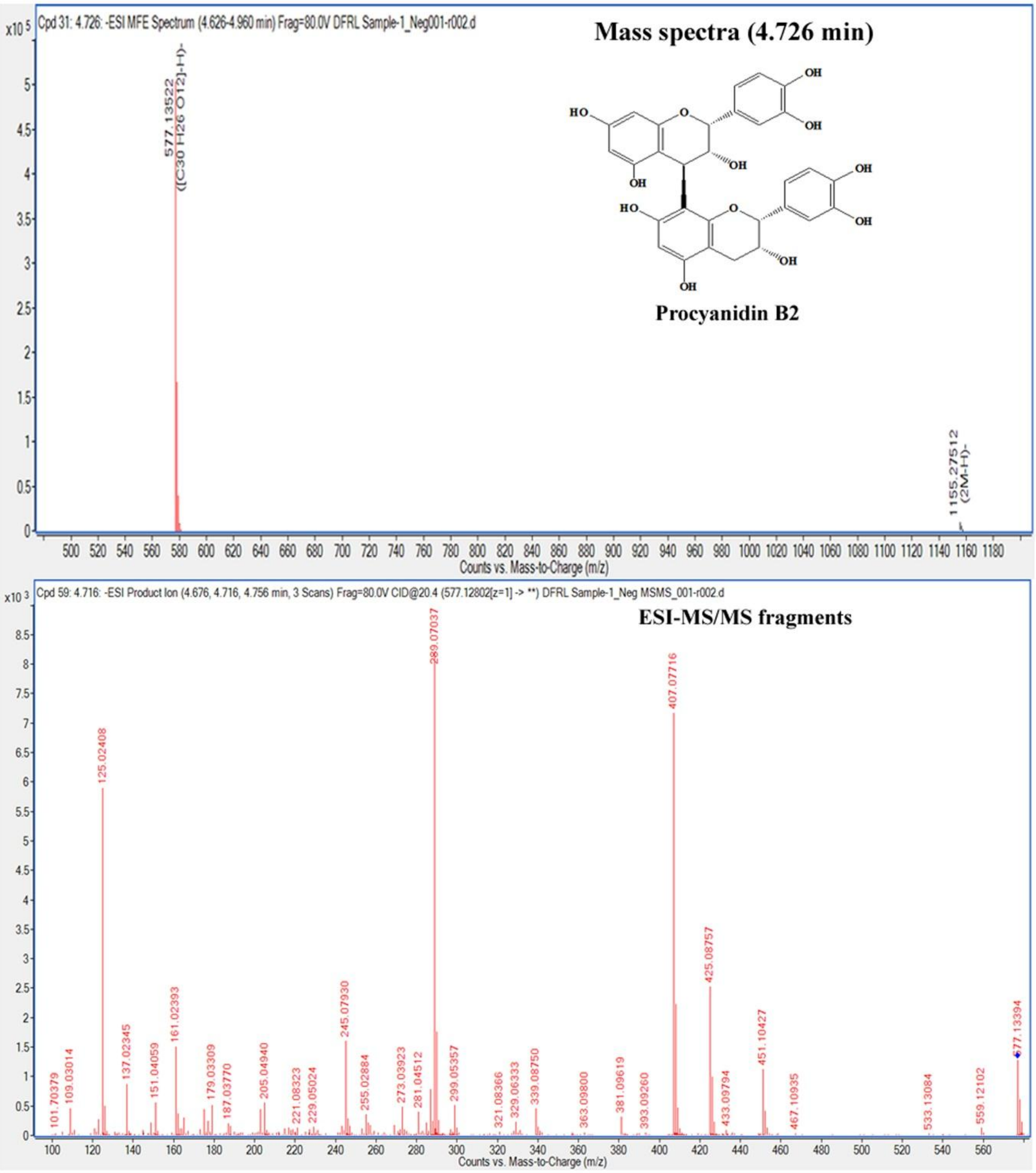

Supplementary Figure S3. LC-MS and ESI-MS/MS profile of TSE showing Procyanidin B2. Peak was obtained at retention time 4.726 minutes. Signal obtained in ESI-MS/MS corresponds to its absolute mass m/z 578.520 daltons. Molecular structure correlator (MSC) software was used to predict the possible structure for the obtained molecular formula and fragmentation pattern. Chemical structure of Procyanidin B2 was drawn by using Chem Draw software.

Supplementary Figure S4

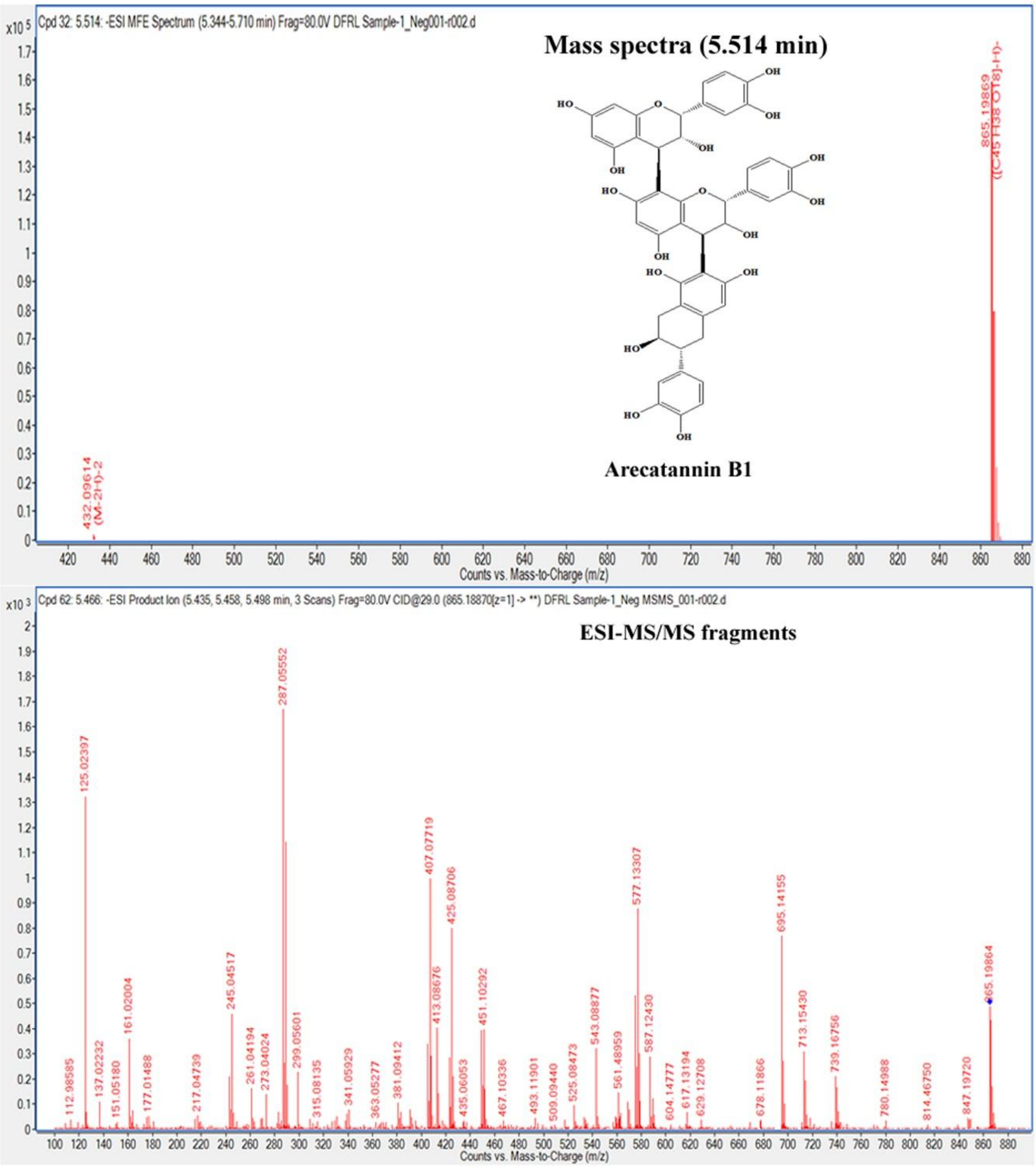

Supplementary Figure S4.LC-MS and ESI-MS/MS profile of TSE showing Arecatannin B1. Peak was obtained at retention time 5.514 minutes. Signal obtained in ESI-MS/MS corresponds to its absolute mass m/z 866.772 daltons. Molecular structure correlator (MSC) software was used to predict the possible structure for the obtained molecular formula and fragmentation pattern. Chemical structure of Arecatannin B1 was drawn by using Chem Draw software.

Supplementary Figure S5

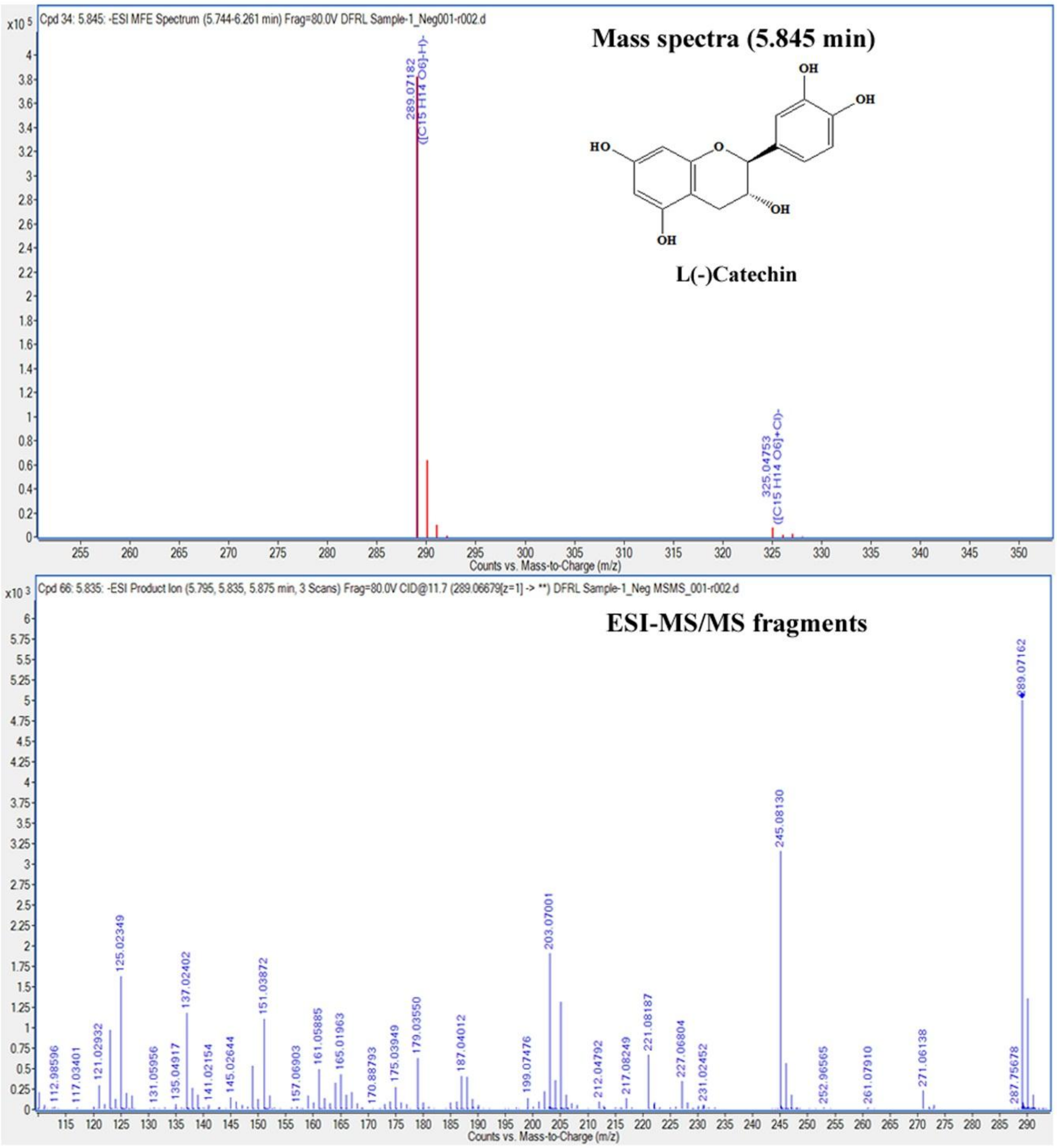

Supplementary Figure S5. LC-MS and ESI-MS/MS profile of TSE showing L(-)Catechin. Peak was obtained at retention time 5.845 minutes. Signal obtained in ESI-MS/MS corresponds to its absolute mass m/z 290.079 daltons. Molecular structure correlator (MSC) software was used to predict the possible structure for the obtained molecular formula and fragmentation pattern. Chemical structure of L(-)Catechin was drawn by using Chem Draw software.

Supplementary Figure S6

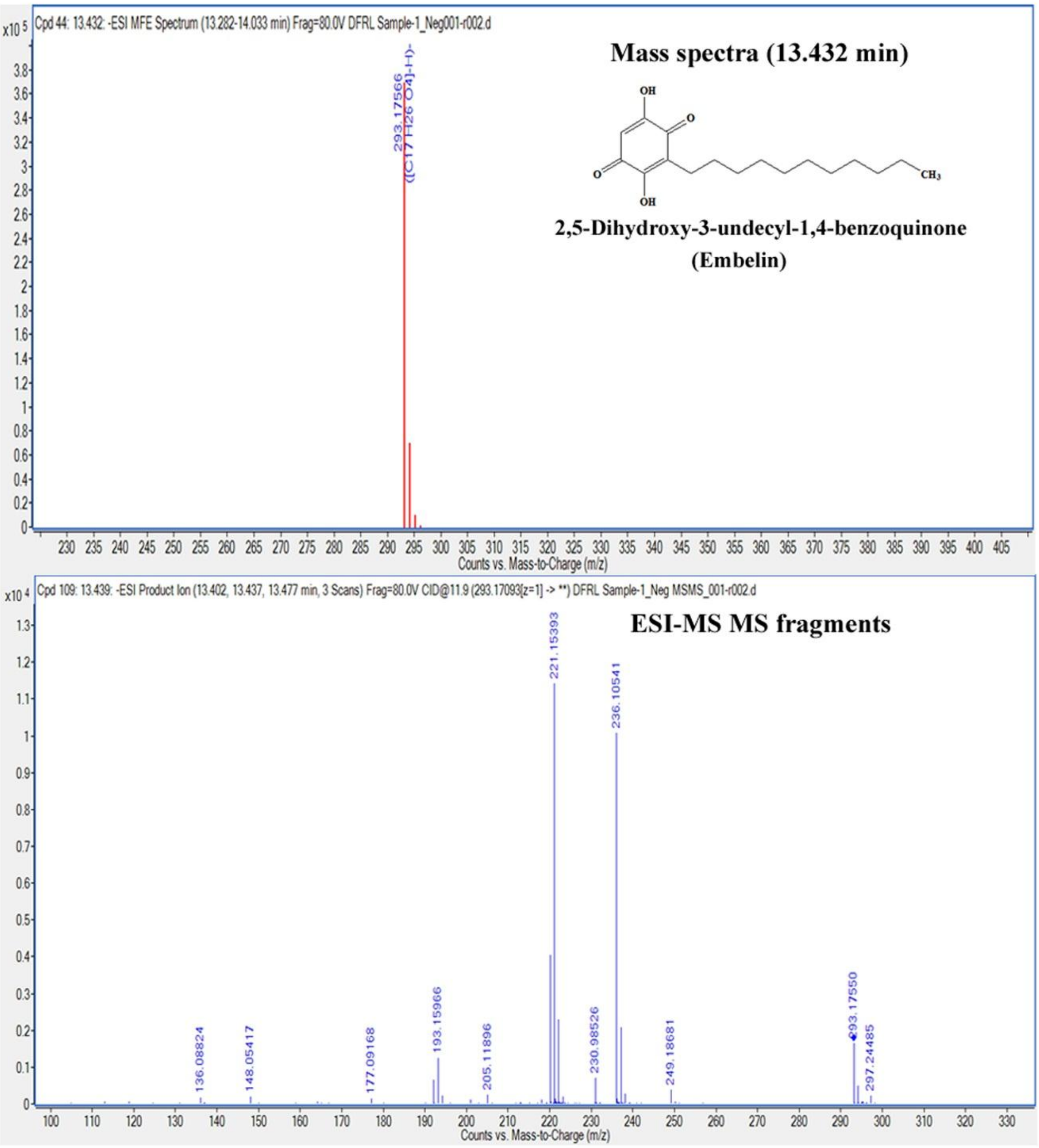

Supplementary Figure S6. LC-MS and ESI-MS/MS profile of TSE showing Embelin. Peak was obtained at retention time 13.432 minutes. Signal obtained in ESI-MS/MS corresponds to its absolute mass m/z 294.183 daltons. Molecular structure correlator (MSC) software was used to predict the possible structure for the obtained molecular formula and fragmentation pattern. Chemical structure of Embelin acid was drawn by using Chem Draw software.

Supplementary Figure S7

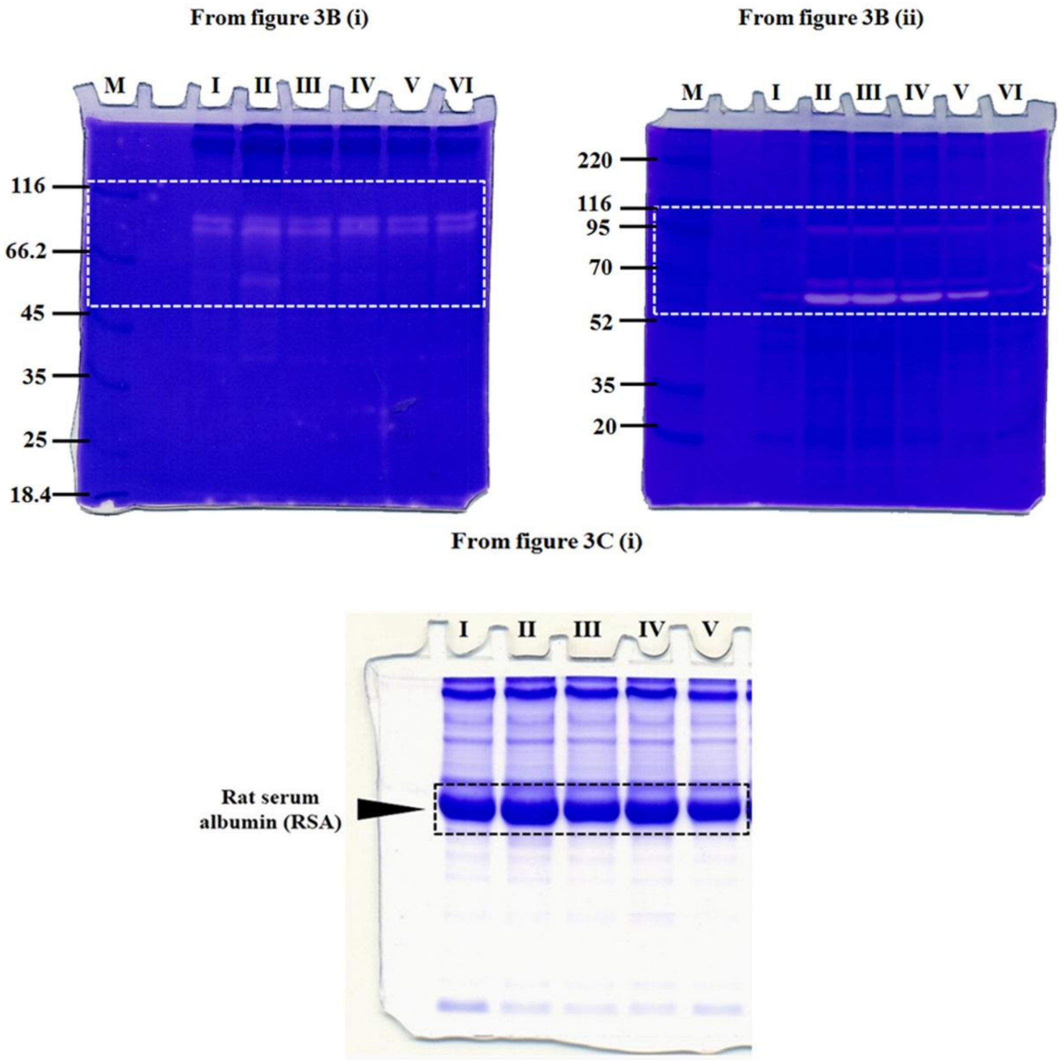

Supplementary Figure S7. Full length images of gelatin zymograms and SDS-PAGE

Full length images of zymograms and SDS-PAGE from figure 3. Regions of interest are highlighted and are presented as cropped images in figure 3. Sample are as follows; Lane I: saline control, Lane II: Arthritic, Lane III: Ibuprofen treated (10 mg/kg), Lane IV: TSE treated (25 mg/kg), Lane V: TSE treated (50 mg/kg) and Lane VI: TSE alone treated (50 mg/kg). M indicates molecular weight markers in kDa.

Supplementary Figure S8

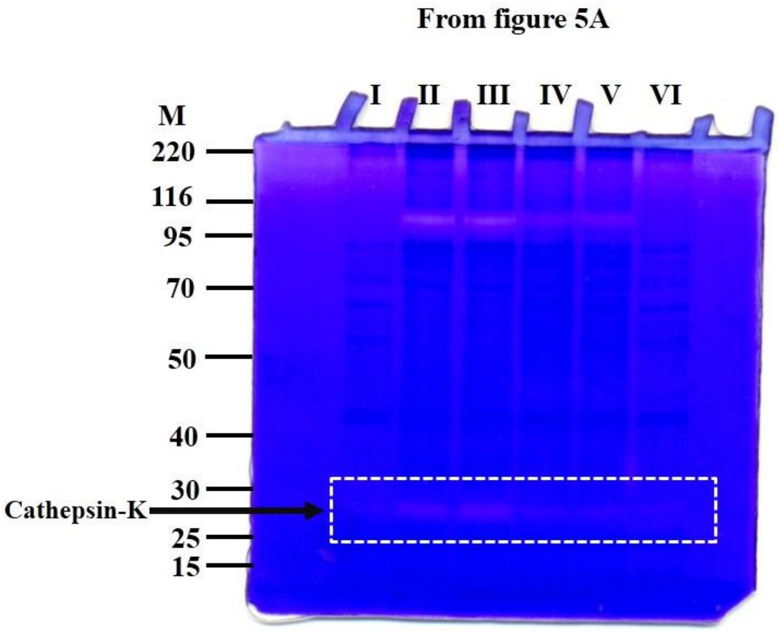

Supplementary Figure S8. Full length image of Cathepsin-K zymogram

Full length image of Cathepsin-K zymogram from figure 5. Regions of interest are highlighted and are presented as cropped image in figure 5. Sample are as follows; Lane I: saline control, Lane II: Arthritic, Lane III: Ibuprofen treated (10 mg/kg), Lane IV: TSE treated (25 mg/kg), Lane V: TSE treated (50 mg/kg) and Lane VI: TSE alone treated (50 mg/kg). M indicates molecular weight markers in kDa.

Supplementary Figure S9

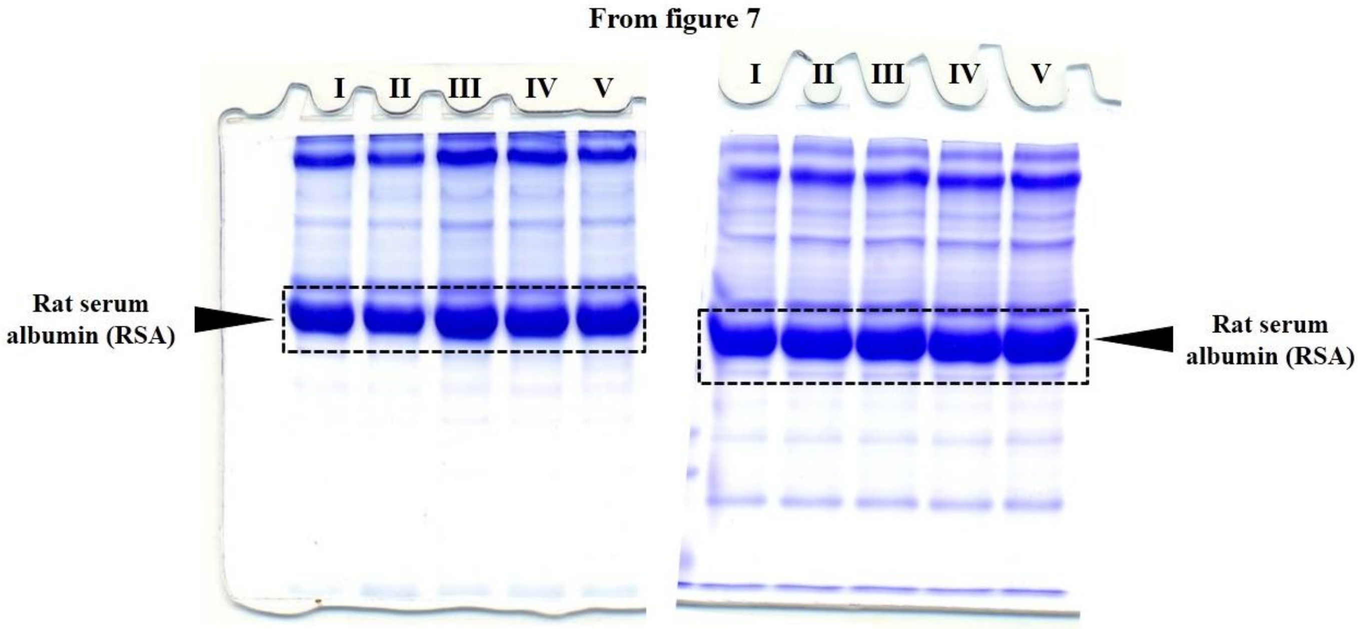

Supplementary Figure S9. Full length image of SDS-PAGE

Full length image of SDS-PAGE from figure 7. Regions of interest are highlighted and are presented as cropped image in figure 7. Sample are as follows; Lane I: saline control, Lane II: Arthritic, Lane III: Ibuprofen treated (10 mg/kg), Lane IV: TSE treated (25 mg/kg) and Lane V: TSE treated (50 mg/kg).
